# Enhanced Mitochondrial Superoxide Scavenging Does Not Improve Muscle Insulin Action in the High Fat-Fed Mouse

**DOI:** 10.1371/journal.pone.0126732

**Published:** 2015-05-19

**Authors:** Daniel S. Lark, Li Kang, Mary E. Lustig, Jeffrey S. Bonner, Freyja D. James, P. Darrell Neufer, David H. Wasserman

**Affiliations:** 1 Department of Molecular Physiology and Biophysics, Vanderbilt University, Nashville, Tennessee, United States of America; 2 Department of Physiology, East Carolina University, Greenville, North Carolina, United States of America; 3 East Carolina Diabetes and Obesity Institute, East Carolina University, Greenville, North Carolina, United States of America; 4 Mouse Metabolic Phenotyping Center, Vanderbilt University, Nashville, Tennessee, United States of America; INSERM/UMR 1048, FRANCE

## Abstract

Improving mitochondrial oxidant scavenging may be a viable strategy for the treatment of insulin resistance and diabetes. Mice overexpressing the mitochondrial matrix isoform of superoxide dismutase (*sod2^tg^* mice) and/or transgenically expressing catalase within the mitochondrial matrix (*mcat^tg^* mice) have increased scavenging of O_2_˙ˉ and H_2_O_2_, respectively. Furthermore, muscle insulin action is partially preserved in high fat (HF)-fed *mcat^tg^* mice. The goal of the current study was to test the hypothesis that increased O_2_˙ˉ scavenging alone or in combination with increased H_2_O_2_ scavenging (*mtAO* mice) enhances *in vivo* muscle insulin action in the HF-fed mouse. Insulin action was examined in conscious, unrestrained and unstressed wild type (WT), *sod2^tg^*, *mcat^tg^* and *mtAO* mice using hyperinsulinemic-euglycemic clamps (insulin clamps) combined with radioactive glucose tracers following sixteen weeks of normal chow or HF (60% calories from fat) feeding. Glucose infusion rates, whole body glucose disappearance, and muscle glucose uptake during the insulin clamp were similar in chow- and HF-fed WT and *sod2^tg^* mice. Consistent with our previous work, HF-fed *mcat^tg^* mice had improved muscle insulin action, however, an additive effect was not seen in *mtAO* mice. Insulin-stimulated Akt phosphorylation in muscle from clamped mice was consistent with glucose flux measurements. These results demonstrate that increased O_2_˙ˉ scavenging does not improve muscle insulin action in the HF-fed mouse alone or when coupled to increased H_2_O_2_ scavenging.

## Introduction

Overnutrition can lead to the development of insulin resistance and type 2 diabetes. Increased production of mitochondrial oxidant species has been proposed as central to the etiology of diet-induced insulin resistance [1]. In the presence of overnutrition, the mitochondrial electron transport system (ETS) is overloaded with electrons donated from reducing equivalents (e.g. NADH and FADH_2_), resulting in the increased production of membrane impermeable superoxide ion (O_2_˙ˉ). In the mitochondrial matrix, O_2_˙ˉ is dismutated by manganese superoxide dismutase (SOD2) to hydrogen peroxide (H_2_O_2_). Both O_2_˙ˉ and H_2_O_2_ have been proposed to serve as metabolic sensors functionally linking redox biology to insulin sensitivity [2, 3]. Increased O_2_˙ˉ scavenging by increased SOD2 activity has been proposed to have beneficial effects on muscle insulin sensitivity. This proposal is complicated by the fact that O_2_˙ˉ scavenging comes at the expense of increased H_2_O_2_ production [4].

Transgenic overexpression of SOD2 (*sod2* ^tg^) is a genetic model of enhanced mitochondrial O_2_˙ˉ scavenging. Overexpression of SOD2 protects against radiation-induced cell death [5], oxygen injury [6], and peroxidative damage to membrane lipids [7]. SOD2 overexpression has also been shown to enhance mitochondrial oxidative capacity [8] and inhibit tumor cell growth [9, 10], effects that are linked to increased production or accumulation of H_2_O_2_ [9, 11, 12]. Mice with transgenic expression of catalase, a peroxisomal enzyme, within the mitochondrial matrix (*mcat* ^tg^) are a model of enhanced mitochondrial H_2_O_2_ scavenging in striated muscle [13]. *mcat* ^tg^ mice have increased lifespan [13] and we have recently demonstrated that *mcat* ^*tg*^ mice are partially protected against diet-induced insulin resistance [3].

**Fig 1:**
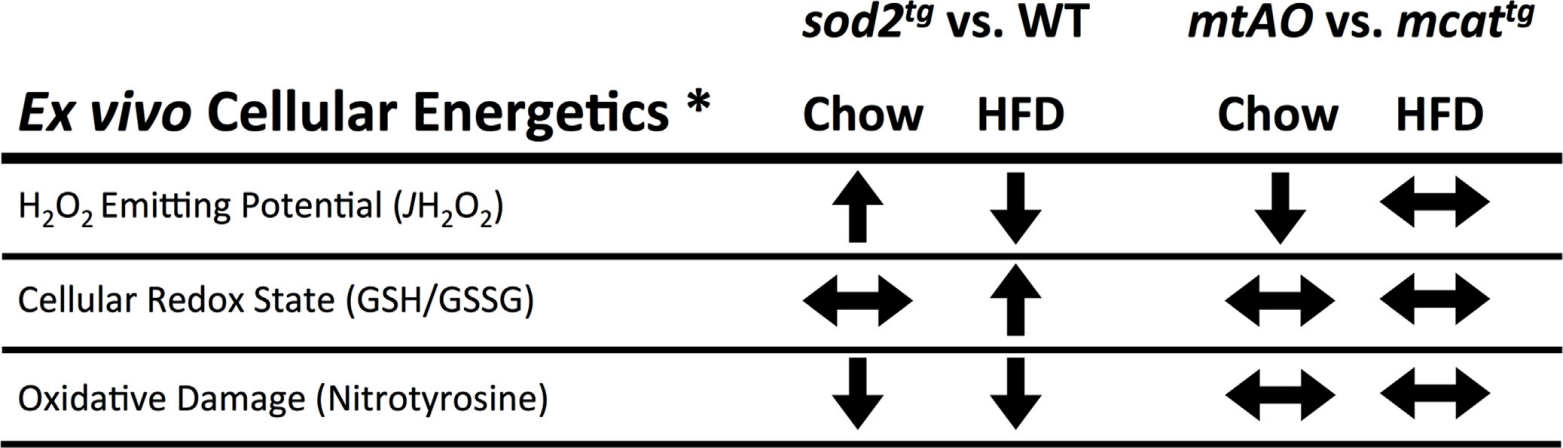
Summary of effects of diet and transgenic expression of SOD2 and/or catalase on oxidant production and cellular redox state. * Data adapted from Kang et al. 2012 [4].

In a previous report, we examined the independent and combined effects of enhanced scavenging of O_2_˙ˉ and H_2_O_2_ in the muscle of HF-fed mice using *sod2* ^*tg*^, *mcat* ^*tg*^ and *mtAO* double transgenic mice generated by crossing *sod2* ^*tg*^ and *mcat* ^*tg*^ [4]. In that report, it was found that cellular redox state (GSH/GSSG), mitochondrial oxidant emitting potential (*J*H_2_O_2_) and oxidative damage (nitrotyrosine accumulation) are differentially altered by HF feeding (Summarized in Fig 1). In particular, HF diet-induced elevations in *J*H_2_O_2_ and oxidation of the cellular redox environment (decreased GSH/GSSG) are ameliorated in both *sod2* ^*tg*^ and *mcat* ^*tg*^ mice. Furthermore, *mtAO* mice have additive protection from HF diet-induced elevations in *J*H_2_O_2_ relative to *mcat* ^*tg*^ mice. Altogether, these findings suggest that enhanced scavenging of O_2_˙ˉ may improve muscle insulin action in the HF-fed mouse.

In the current report, we tested the hypothesis that enhanced mitochondrial O_2_˙ˉ scavenging improves insulin action in HF-fed mice. To assess insulin action, hyperinsulinemic-euglycemic clamps (insulin clamp) combined with isotopic glucose tracers were performed in conscious, unrestrained and unstressed animals. This hypothesis was tested in *sod2* ^tg^ mice with normal H_2_O_2_ scavenging capacity and mtAO mice with enhanced H_2_O_2_ scavenging capacity.

## Methods and Materials

### Animals and Ethical Approvals

All mice were on the C57BL/6J background and housed in cages under conditions of controlled temperature and humidity with a 12-h light/dark cycle. As we have done previously [4], *sod2* ^tg^ mice with ~3 fold overexpression of SOD2 in muscle were bred with *mcat* ^tg^ mice generously provided by Dr. Peter Rabinovitch of the University of Washington [13]. This breeding scheme generated four distinct genotypes: wild type (WT), *sod2* ^*tg*^, *mcat* ^*tg*^ and double transgenic (*mtAO)* mice. All mice were fed either a chow or HF diet (F3282, BioServ), which contains 60% calories as fat, for 16 weeks. Body composition was determined using an mq10 nuclear magnetic resonance analyzer (Brucker Optics). The Vanderbilt University Medical Center and the East Carolina University Animal Care and Use Committees approved all animal procedures.

### Hyperinsulinemic-Euglycemic Clamp

In preparation for a hyperinsulinemic-euglycemic, hereafter referred to as an insulin clamp, mice had catheters implanted in the left carotid artery and the right jugular vein for blood sampling and intravenous infusion, respectively, at least 5 days prior to the study [14]. The insulin clamp procedure used in these studies is unique in that it has been validated from a number of standpoints including: stability of blood glucose over time, absence of stress (no increase in catecholamine concentrations), insulin levels and absence of a fall in hematocrit [14]. The entire insulin clamp procedure has been visually published [15] and described in depth [14, 16] previously. Readers are also referred to a publicly available guide to the surgery and clamp procedure that is maintained by the Vanderbilt Mouse Metabolic Phenotyping Center, found at: (http://www.mc.vanderbilt.edu/documents/mmpc/files/2012%20Lab%20Manual(1).pdf).

In the current study, the insulin clamp coupled with isotopic tracer techniques was performed in a total of 75 conscious, unstressed mice. Mice were fasted for 5 hours beginning at ~ 7 am on the day of the insulin clamp. Ninety minutes prior to starting the insulin infusion (-90 min), [3-^3^H]glucose was primed (2.4 μCi) and continuously infused at 0.04 μCi/min into the jugular vein catheter and then increased to 0.12 μCi/min during the insulin clamp. Baseline blood or plasma parameters were determined as the mean of values obtained in arterial blood collected at -15 and -5 min. At time 0, infusion of human insulin (4 mU/kg/min) was started and continued for the entire clamp (155 min). This amount of insulin is a high, but physiological, concentration that was used to ensure insulin stimulation in tissues of HF-fed mice but allow for discrimination of differences between groups. During the insulin infusion, blood glucose was clamped at ~150 mg/dL by a variable infusion of exogenous glucose (50% dextrose). Mice received heparinized saline-washed erythrocytes from donor mice at 5 μL/min to prevent a fall of hematocrit. Blood glucose was monitored and the glucose infusion rate (GIR) was adjusted every 10 min throughout the clamp. Whole blood samples (~ 50 μl) were taken from 80–120 min for the determination of plasma [3-^3^H]glucose. Blood samples were taken to measure plasma insulin at t = 100 and 120min. At 120min, 13μCi of 2-[^14^C]deoxyglucose ([^14^C]2-DG) was administered as an intravenous bolus. Blood samples were then taken at 2, 15, 25, and 35min following the bolus for the determinations of plasma [^14^C]2-DG levels. After the last blood sample was taken, mice were anesthetized with phenobarbital and tissues were removed and frozen in liquid nitrogen.

### Processing of Plasma and Tissue Samples

Plasma insulin was determined using an insulin ELISA kit (Millipore). Non-esterified fatty acid (NEFA) concentrations were measured using an enzymatic colorimetric assay (NEFA C kit, Wako Chemicals). Radioactivity of plasma and tissue samples was measured as previously described [14].

Glucose appearance (Ra) and disappearance (Rd) rates were calculated using non-steady state equations [17]. Endogenous glucose production (EndoRa) was determined by subtracting the glucose infusion rate from total Ra. A glucose metabolic index for tissues (R_g_) was calculated as described previously [18].

### Western Blotting

Gastrocnemius muscle samples were homogenized in buffer containing (pH = 7.5): 50 mM Tris, 1 mM EDTA, 1 mM EGTA, 10% glycerol, 1% Triton X-100, 1 mM DTT, 1 mM PMSF, 5 μg/mL protease inhibitor cocktail, 50 mM NaF, 5 mM sodium pyrophosphate, and centrifuged at 13,000xg for 20min at 4°C. The protein concentration was then determined and homogenates were run on SDS-PAGE gels. Phosphorylated and total Akt/PKB were probed using phospho-Akt (Ser^473^) and Akt primary antibodies (Cell Signaling) and IRdye 800CW secondary antibodies (LI-COR Biosciences). Band densities were quantified using Odyssey (LI-COR Biosciences) software.

### Statistical Analysis

Data are expressed as mean ± SEM. Statistical comparisons were made between WT and *sod2* ^*tg*^ mice or *mcat* ^*tg*^ and mtAO mice using unpaired student’s t-test or two-way ANOVA with Tukey post-hoc test when appropriate. The significance level was set at *p*<0.05.

## Results

### Basal metabolic characteristics

WT, *sod2* ^*tg*^, *mcat* ^*tg*^, and *mtAO* mice were fed either normal chow or a HF diet for 16 weeks and studied at 19 weeks of age. On normal chow, no genotype differences were observed in body weight; however, *mtAO* mice displayed lower fasting glucose and insulin compared to *mcat* ^*tg*^ littermates (Table 1). HF feeding increased body weight, basal 5-h fasting glucose and insulin as expected in all genotypes and was not affected by SOD2 overexpression. Basal arterial NEFA levels were not altered by genotype or diet. The percentage of body fat was increased by HF feeding, but not affected by genotype.

**Table 1:** Basal (5h-fasted) and insulin clamp characteristics of *sod2* ^*tg*^, *mcat* ^*tg*^, and *mtAO* mice.

	Chow		HF Diet		Chow		HF Diet	
	WT	*sod2* ^*tg*^	WT	*sod2* ^*tg*^	*mcat* ^*tg*^	*mtAO*	*mcat* ^*tg*^	*mtAO*
n (female/male)	11 (4/7)	9 (4/5)	11 (5/6)	10 (4/6)	6 (3/3)	7 (4/3)	11 (6/5)	10 (5/5)
Body weight (g)	24 ± 1	25 ± 1	36 ± 2^†^	35 ± 2^†^	23 ± 2	24 ± 1	33 ± 2^†^	33 ± 2^†^
Fat Mass (%)	9 ± 1	10 ± 1	30 ± 2^†^	33 ± 4^†^	9 ± 1	9 ± 1	28 ± 3^†^	26 ± 5^†^
*Arterial Glucose (mg∙dL* ^*-1*^)								
Basal	121 ± 6	129 ± 5	158 ± 8^†^	150 ± 4^†^	132 ± 8	110 ± 7^§^	134 ± 5	142 ± 5^†^
Insulin clamp ^ƒ^	156 ± 3	149 ± 6	155 ± 3	147 ± 7	151 ± 6	156 ± 2	159 ± 3	155 ± 3
*Insulin (ng∙mL* ^*-1*^)								
Basal	0.7 ± 0.1	0.8 ± 0.1	1.8 ± 0.5^†^	1.5 ± 0.4^†^	0.8 ± 0.1	0.4±0.1^§^	1.6 ± 0.3^†^	1.5 ± 0.3^†^
Insulin clamp ^ƒ^	2.8 ± 0.5	2.8 ± 0.3	3.5 ± 0.3	3.6 ± 0.4	3.4 ± 0.7	3.6 ± 0.6	3.7 ± 0.4	3.0 ± 0.3
*NEFA (mM)*								
Basal	0.9±0.10	1.0±0.06	0.7±0.04	0.8±0.08	0.9±0.08	0.8±0.06	0.7±0.04	0.7±0.03
Insulin clamp ^ƒ^	0.4±0.05	0.5±0.04	0.5±0.05	0.4±0.03	0.4±0.03	0.3±0.02	0.4±0.03	0.4±0.04

^ƒ^ Data are presented as the average of values obtained from 80–120 min during the hyperinsulinemic-euglycemic clamp.

^†^ *p*<0.05 compared to Chow within the same genotype.

^§^ *p*<0.05 compared to Chow *mcat* ^*tg*^.

All data are expressed as mean ± SEM.

### Hyperinsulinemic-euglycemic clamps

**Fig 2:**
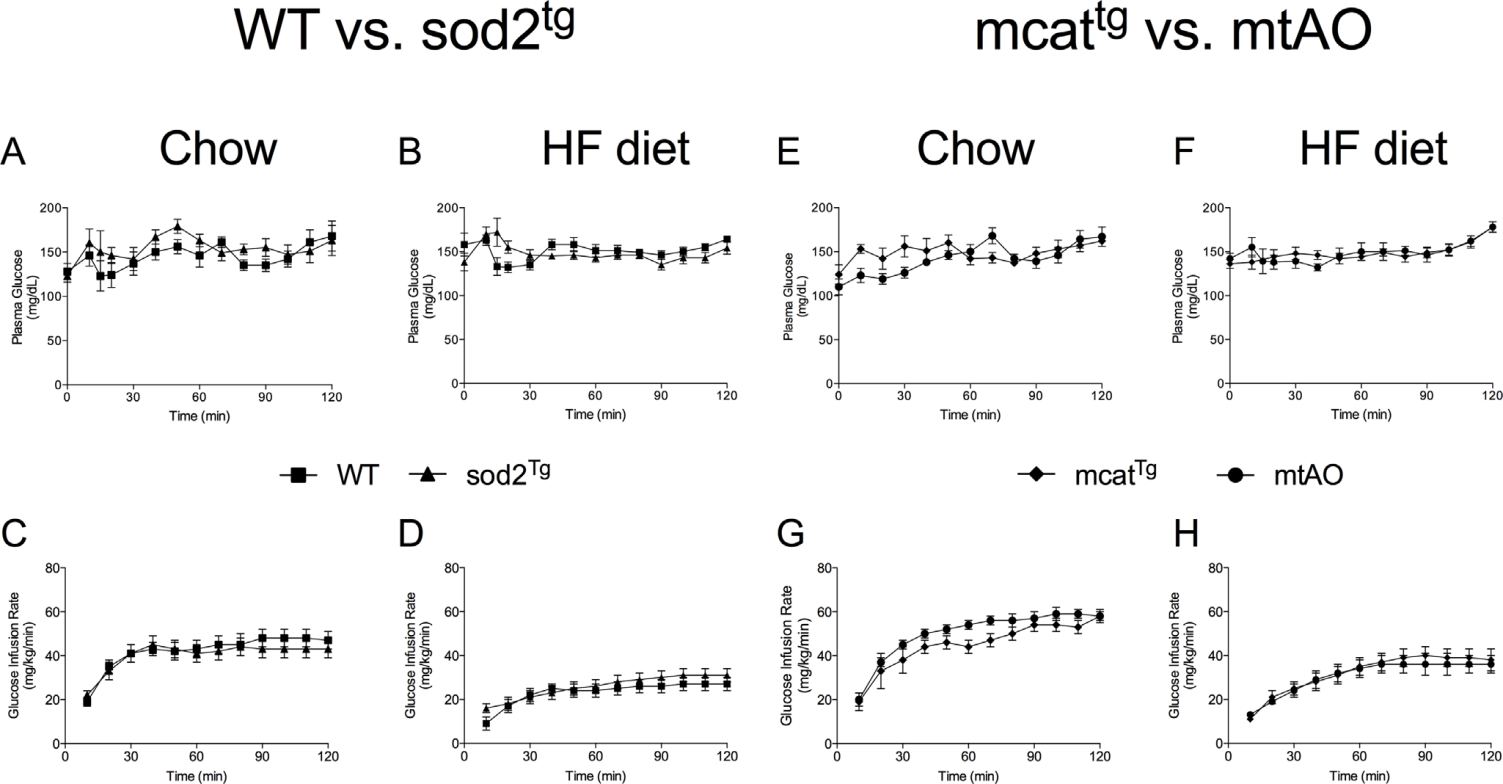
SOD2 overexpression does not augment glucose infusion rates in HF-fed mice during an insulin clamp. (**A-D**) Blood glucose was maintained at ~150 mg/dL in all groups (diet and genotype) during the insulin clamp by variable venous infusion of 50% glucose. (**E-H**) Glucose infusion rate (GIR) over the 120-minute duration of the insulin clamp.

Blood glucose was maintained at ~150 mg/dL during the insulin clamp in all groups of mice using a variable glucose infusion (Table 1, Fig 2A, 2B, 2E and 2F). Infusion of insulin (4 mU/kg/min) increased plasma insulin to a similar extent in all groups of mice (Table 1). Insulin-induced suppression of arterial NEFA was not affected by genotype. HF feeding decreased glucose infusion rate (GIR) in all four genotypes relative to chow, but, consistent with our previous findings [3], GIR was partially protected in HF-fed *mcat* ^*tg*^ mice compared to WT (average GIR during steady state: WT -27.18 ± 3.335, *mcat* ^*tg*^ -40.55 ± 4.424, *p<0*.*05*). GIR was not improved in chow or HF-fed *sod2* ^*tg*^ or *mtAO* mice compared to WT (Fig 2C and 2D) or *mcat* ^*tg*^ (Fig 2G and 2H) mice, respectively.

**Fig 3:**
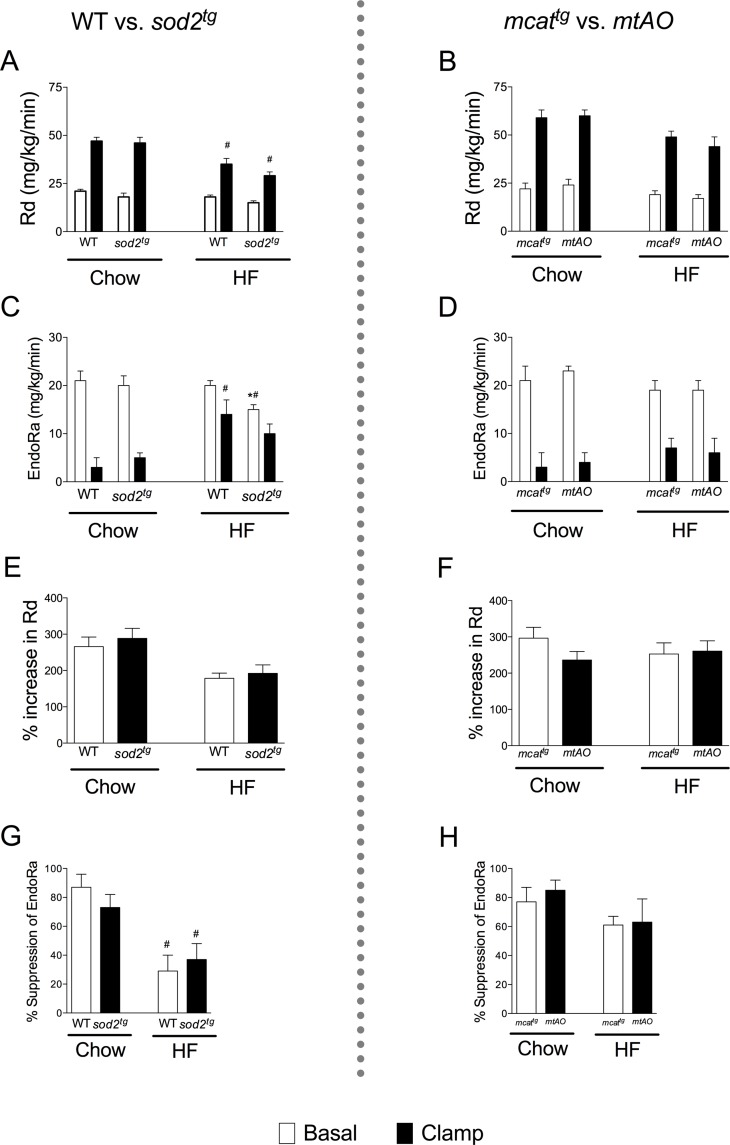
Rates of endogenous glucose production (EndoRa) and glucose disappearance (Rd) during the insulin clamp. Rd was calculated using the tracer [3-^3^H]glucose dilution method in SOD2 overexpressing WT (**A**) and *mcat* ^*tg*^ (**B**) mice. EndoRa was determined by subtracting the glucose infusion rate from total Ra in sod2 overexpressing WT (**C**) and *mcat* ^*tg*^ (**D**) mice. Insulin-stimulated increase in Rg (**E and F**) and suppression of EndoRa (**G and H**) are presented as percentage (%) of basal rates. *p<0.05 compared with *sod2* ^*tg*^ overexpressing mice (WT or *mcat* ^*tg*^) within a diet; ^#^ *p*<0.05 compared with chow within a genotype.

HF diet-induced impairment of clamp Rd was not attenuated in *sod2* ^*tg*^ compared to WT (Fig 3A) or *mtAO* in relation to *mcat* ^*tg*^ (Fig 3C). Basal, but not insulin-suppressed, endoRa was decreased in HF-fed *sod2* ^*tg*^ mice, but not chow-fed *sod2* ^*tg*^ mice compared to WT (Fig 3B). EndoRa was not affected under basal or insulin clamp conditions in chow or HF-fed *mtAO* mice (Fig 3D). The percentage increase in Rg (Fig 3E and 3F) and suppression of EndoRa (Fig 3G and 3H) in response to insulin were not altered by SOD2 overexpression.

**Fig 4:**
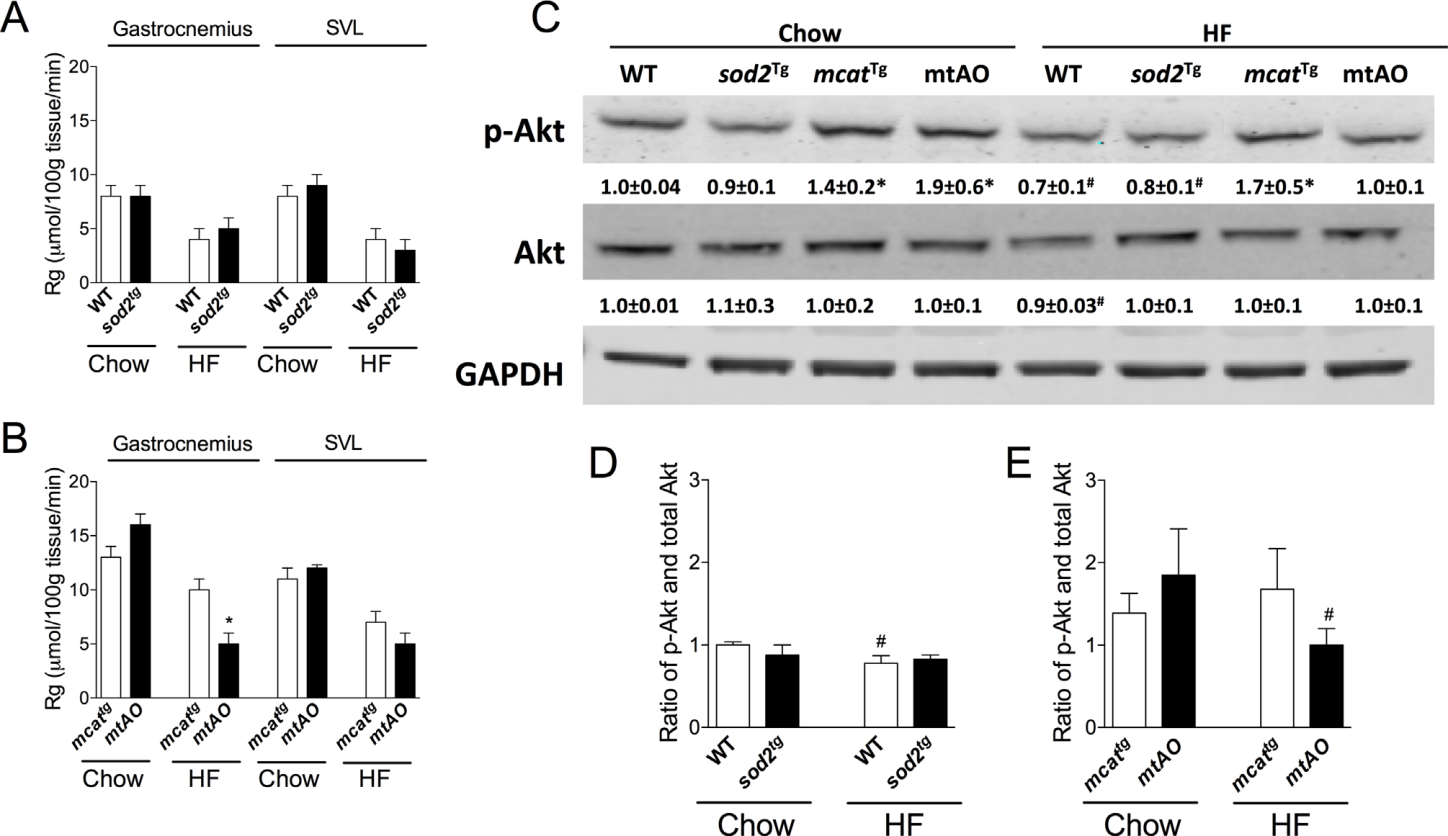
Divergent effects of SOD2 overexpression on muscle glucose uptake (Rg) and insulin signaling in HF-fed WT and *mcat* ^*tg*^ mice. Non-metabolizable glucose analog [^14^C]2-deoxyglucose was administered as an intravenous bolus to determine muscle glucose uptake (Rg) using liquid scintillation counting in SOD2 overexpressing WT (**A**) and *mcat* ^*tg*^ (**B**) mice. Insulin signaling was measured in tissue homogenates extracted from gastrocnemius, applied to 4–12% SDS-PAGE gel and Western blotted with anti-phospho-Akt (Ser^473^) or anti-total Akt antibodies. Values are expressed as mean ± SEM of integrated intensity and representative bands are presented (**C**). Ratio of phosphorylated-Akt to total Akt was calculated in SOD2 overexpressing WT (**D**) and mcat^tg^ (**E**) mice. n = 4–6. *p<0.05 compared with *sod2* ^*tg*^ or WT within a diet; ^#^ *p*<0.05 compared with chow within a genotype. SVL, superficial vastus lateralis.

SOD2 overexpression did not augment Rg in chow- or HF-fed WT (Fig 4A) mice. Gastrocnemius Rg in normal chow-fed *mtAO* mice was not changed but was decreased in HF-fed *mtAO* mice compared to *mcat* ^*tg*^ (Fig 4B). SVL Rg in *mtAO* and *mcat* ^*tg*^ mice was not different (**data not shown**).

### Insulin signaling

Insulin signaling was assessed in gastrocnemius by measuring the ratio of phosphorylated to total expression of Akt, a hub of the insulin signaling cascade (Fig 4C). The ratio of phosphorylated to total Akt was not altered in chow- or HF-fed *sod2* ^*tg*^ mice (Fig 4D). However, the ratio of phosphorylated to total Akt was decreased in HF-fed mtAO compared to *mcat* ^*tg*^ mice (Fig 3E). Overall Akt phosphorylation was consistent with observed differences in muscle glucose uptake.

## Discussion

Genetic models that modulate the cellular redox environment, like those used in the present study, may provide insight into targets that could be used to treat insulin resistance and diabetes. In this report, we tested the hypothesis that increased O_2_˙ˉ scavenging capacity by SOD2 overexpression would prevent muscle insulin resistance in HF-fed mice. The data provided do not support this hypothesis as enhanced scavenging of O_2_˙ˉ failed to improve muscle insulin action *in vivo*. This conclusion is based on findings in both *sod2* ^*tg*^ mice and *mtAO* mice that have enhanced scavenging of both O_2_˙ˉ and H_2_O_2_. The current study made conclusions based on evaluations of both male and female mice with no regard for estrous cycle. There is considerable evidence that female sex hormones (e.g. estrogen and progesterone) impact insulin sensitivity (reviewed by [19]). Moreover, progesterone, but not estrogen, has been demonstrated to increase mitochondrial oxidant production in non-menopausal women [20]. Therefore, the unknown contribution of these factors is a caveat to the findings presented here.

Previous studies have demonstrated that HF-fed *sod2* ^tg^ mice display improved glucose [2, 21] and insulin tolerance [2]. Glucose tolerance is determined by the insulin secretory response, insulin clearance, non-insulin-mediated glucose disposal, and insulin-mediated glucose disposal [22]. Notably, numerous studies have shown that oxidative stress impairs insulin secretion [23, 24]. The increased oxidative burden incurred by overexpression of SOD2 in the pancreas would be predicted to cause glucose intolerance independent of changes in muscle insulin action. The measurement of insulin tolerance is also complicated by numerous factors including hypoglycemic counterregulation and the variable stimulus due to a spike of insulin [22]. SOD2 overexpressing HF-fed mice were reported to have a very small improvement on insulin tolerance compared to WT (~15%), but still remained considerably impaired in relation to the insulin tolerance in lean mice [2]. The present study, in which we clearly show that insulin action is unaffected by SOD2 overexpression, is largely consistent with the previous measurement of insulin tolerance. Moreover, our finding complements recent work demonstrating that heterozygous knockout of SOD2 does not impair insulin action in chow- or HF-fed mice [25]. Notably, overexpression of sod2 in skeletal muscle using *in vivo* electroporation has been shown to enhance glucose uptake in the HF-fed rat [26]. However, it should be noted that sod2 overexpression in this report was accompanied by an increase in the activity of glutathione peroxidase, which catalyzes the breakdown of H_2_O_2_, and a decrease in protein carbonylation. Thus, the net effect of increased sod2 expression in this model was reduction, not oxidation, of the cellular redox environment. Therefore, we contend that the enhanced insulin sensitivity observed by sod2 overexpression in this report was a consequence of enhanced scavenging of H_2_O_2_, not O_2_˙ˉ.

**Fig 5:**
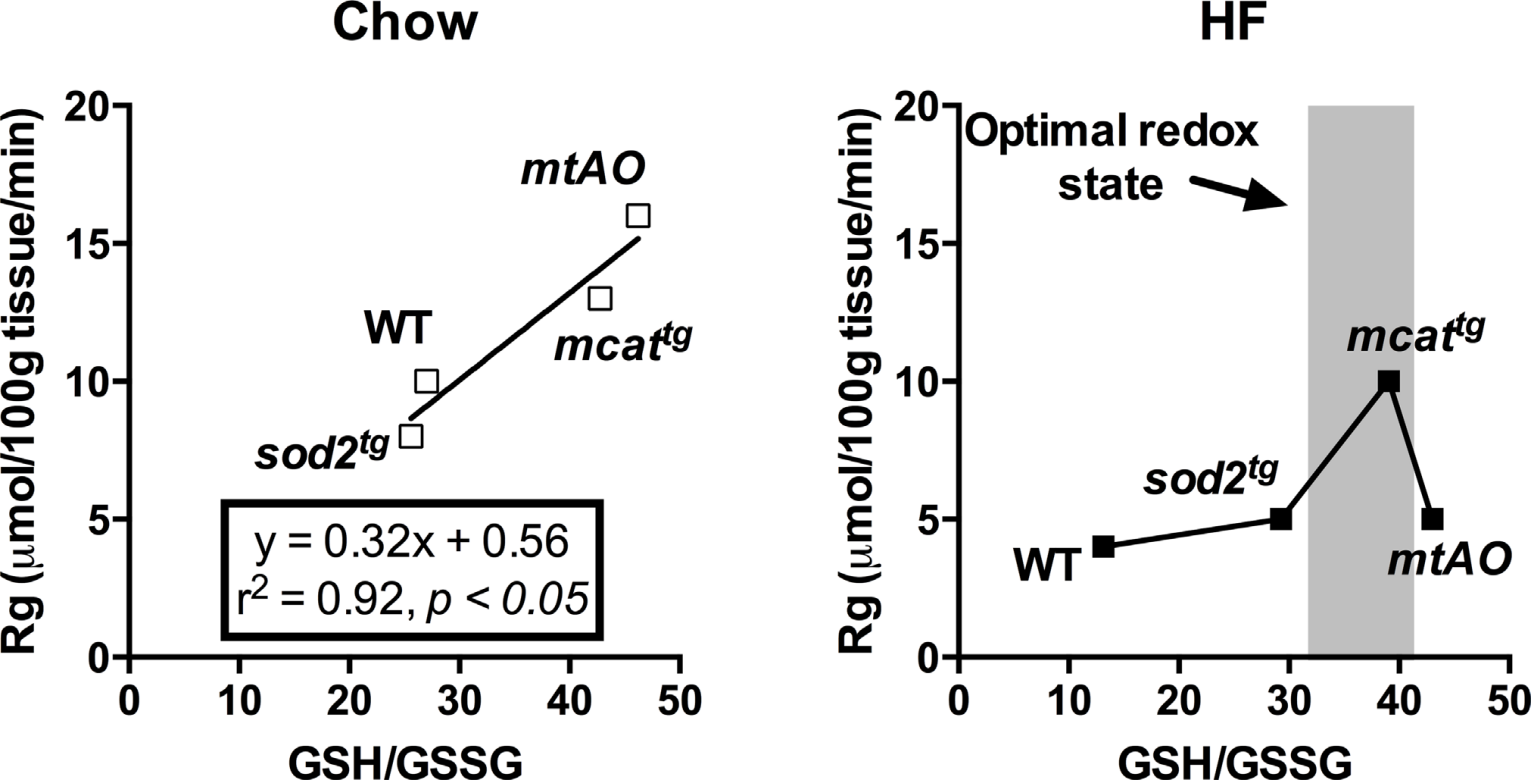
The relationship between glutathione redox state and muscle glucose uptake in chow and HF-fed mice. Rg determined by 2[^14^C]deoxyglucose during the insulin clamp plotted as a function of glutathione redox state (GSH/GSSG) in gastrocnemius of chow- (**A**) and HF-fed (**B**) mice. GSH/GSSG values were adapted from Kang et al. [4].

Previous work has demonstrated that H_2_O_2_ can either increase [27] or decrease [28, 29] skeletal muscle insulin signaling and glucose uptake. H_2_O_2_-mediated impairments in insulin signaling are associated with a selective loss of insulin receptor substrate (IRS)-1 and IRS-2 proteins, in part related to a p38 mitogen-activated protein kinase-dependent mechanism [30]. A recent paper has demonstrated that both *mcat* ^tg^ and *mtAO* mice are protected against oxidation of the cellular glutathione pool on both chow and HF diet [4] (Fig 1). Importantly, when considered in the context of the current report, these previous findings reveal a paradoxical situation where, despite a reduced cellular redox environment, combined enhancement of O_2_˙ˉ and H_2_O_2_ scavenging fails to improve muscle insulin action in response to HF feeding. Consistent with recently reported findings [28–30], plotting Rg obtained in the current study as a function of glutathione redox state (GSH/GSSG) in the skeletal muscle of mice from our previous study [4] reveals two distinct relationships. First, that Rg is positively correlated with insulin-stimulated Rg in chow-fed mice (Fig 5A). However, this linear relationship is incompatible in mice fed a HF diet because insulin-stimulated Rg is low when GSH/GSSG exists above or below a potentially “critical” range (Fig 5B). These results altogether support a paradigm where low rates of mitochondrial H_2_O_2_ production (*J*H_2_O_2_) play a role in maintaining cellular homeostasis (i.e., prevents excessive reduction of cellular redox environment) while a sustained elevation in mitochondrial *J*H_2_O_2_ shifts the cellular redox environment to a more oxidized state and worsens muscle insulin action [31].

## Conclusions

The current report demonstrates that SOD2 overexpression does not alleviate muscle insulin resistance even when combined with increased scavenging of its reaction product, H_2_O_2_. This study, combined with our earlier work using *mcat* ^*tg*^ mice [3, 4], demonstrate that drugs designed to mitigate H_2_O_2_ production and/or enhance H_2_O_2_ scavenging will have a greater benefit for the treatment of diabetes and insulin resistance than those that target O_2_˙ˉ.
